# Supplementary material for: Study of the Phytoextraction and Phytodegradation of Sulfamethoxazole and Trimethoprim from Water by Limnobium laevigatum
Source: Int J Environ Res Public Health. 2022 Dec 17;19(24):16994. doi: 10.3390/ijerph192416994 (PMC9779370; doi:10.3390/ijerph192416994)
Supplement: Supplementary file 1 [file ijerph-19-16994-s001.zip › ijerph-2096964-supplementary.pdf]

Study of the phytoextraction and phytodegradation of sulfamethoxazole and trimethoprim from water by *Limnobium laevigatum*

Klaudia Stando[1,*], Aleksandra Czyż[1], Magdalena Gajda[1], Ewa Felis[2,3], Sylwia Bajkacz[1,2]

[1] *Silesian University of Technology, Faculty of Chemistry, Department of Inorganic, Analytical Chemistry and Electrochemistry, B. Krzywoustego 6 Str., 44-100 Gliwice, Poland*

[2] *Silesian University of Technology, Biotechnology Centre, B. Krzywoustego 8 Str., 44-100 Gliwice, Poland*

[3] *Silesian University of Technology, Faculty of Power and Environmental Engineering, Environmental Biotechnology Department, Akademicka 2 Str., 44-100 Gliwice, Poland*

SUPPLEMENTARY MATERIAL

Summary

This supporting information file includes additional results and information as described in the text of the main article including:

Table S1. Structures of identified SMX and TRI transformation products

Figure S1. Representative LC-MS/MS chromatograms of the $[M+H]^+$ions: TRI (m/z 291.2), (2) SMX (m/z 254.2)

Table S1. *Structures of identified SMX and TRI transformation products*

Abbrevation	Q_1 (m/z)	Q_3 (m/z)	Formula	Structure	Reference
**TRI**	291.2	123.1<br>230.1	$C_{14}H_{18}N_4O_3$	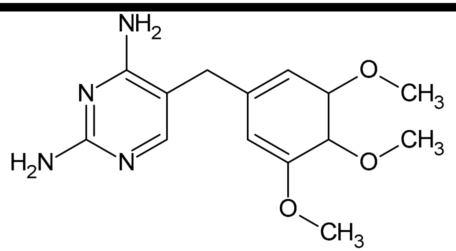	-
TRI171	171.0	126.0<br>81.0	$C_4H_3N_4O_4^+$	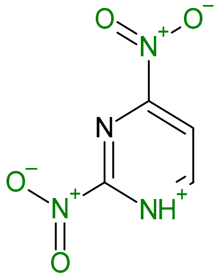	[1]
TRI325	325.0	307.1<br>181.0	$C_{14}H_{20}N_4O_5$	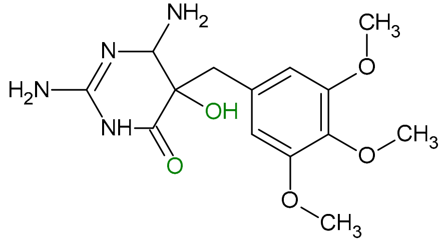<br>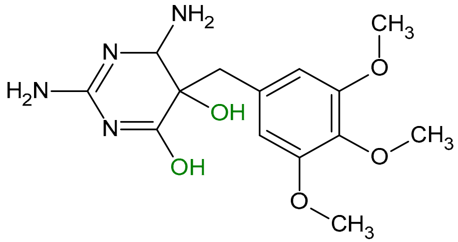	[2,3]

TRI291	291.1	258.1<br>261.2<br>231.1	$C_{13}H_{14}N_4O_4$	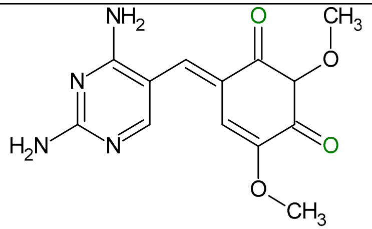<br>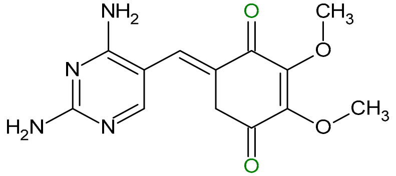<br>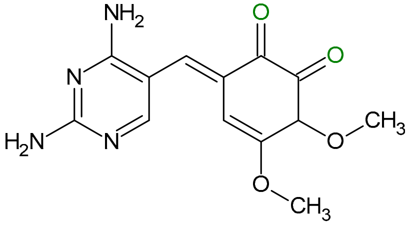<br>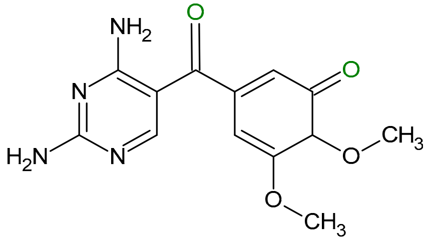<br>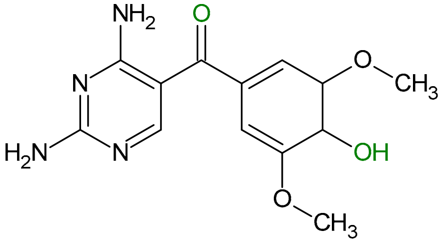	[2]

TRI323	323.1	231.1	$C_{14}H_{18}N_4O_5$	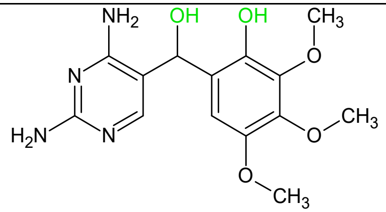 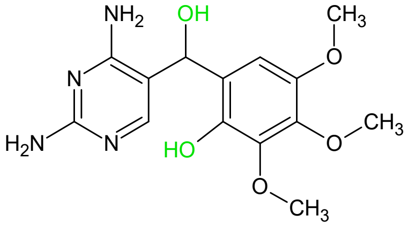 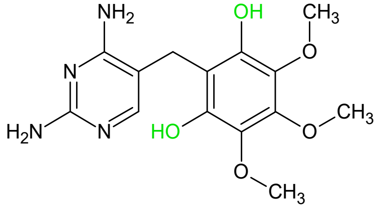	[4]
TRI305	305.2	275.0	$C_{14}H_{16}N_4O_4$	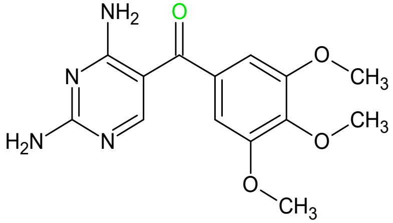	[5]

TRI307	307.1	259.1	$C_{14}H_{18}N_4O_4$	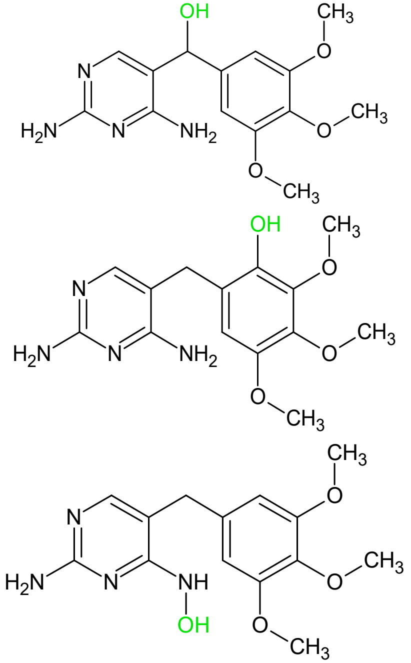	[2]
SMX	254.2	108.1<br>156.0	$C_{10}H_{11}N_3O_3S$	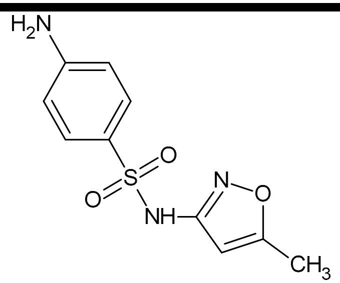	-
SMX173	173.0	156.0<br>108.0<br>94.0	$C_6H_8N_2O_2S$	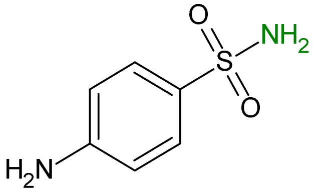	[6]

SMX256	256.1	214.1<br>173.0	$C_{10}H_{14}N_3O_3S$	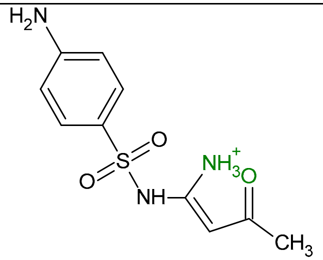	[7]
SMX288	288.0	156.0<br>108.0<br>270.0	$C_{10}H_{13}N_3O_5S$	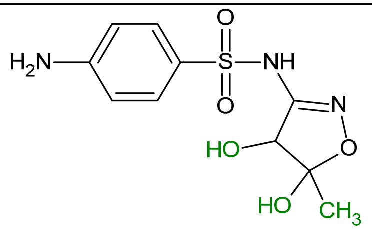	[6,8–10]
SMX304	304.1	286.9<br>275.0	$C_{10}H_{10}N_3O_6S$	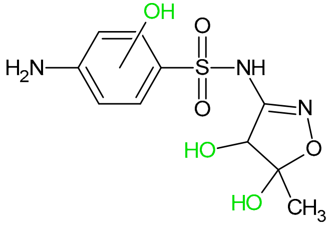	This work
SMX254	254.0	156.0<br>108.0	$C_{10}H_{11}N_3O_3S$	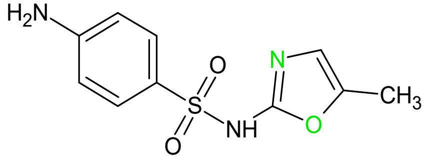	[8]
SMX445	445.1	148.0<br>190.0	$C_{20}H_{25}N_6O_4S$	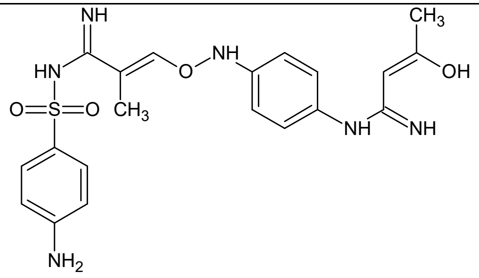	[7]

SMX216	216.0	156.0<br>92.0	$C_7H_9O_3N_3S$	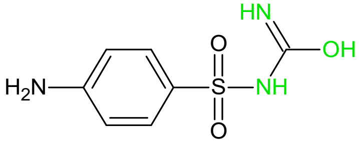	[6]
SMX179	179.4	161.0<br>105.8	$C_4H_6N_2O_4S$	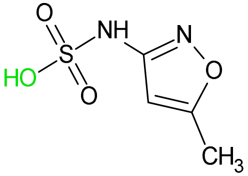	[8]
SMX246	246.0	156.0<br>92.0	$C_8H_{12}N_3O_4S$	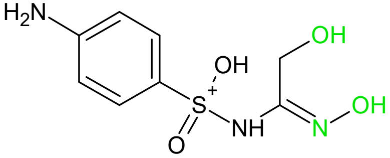	[7]

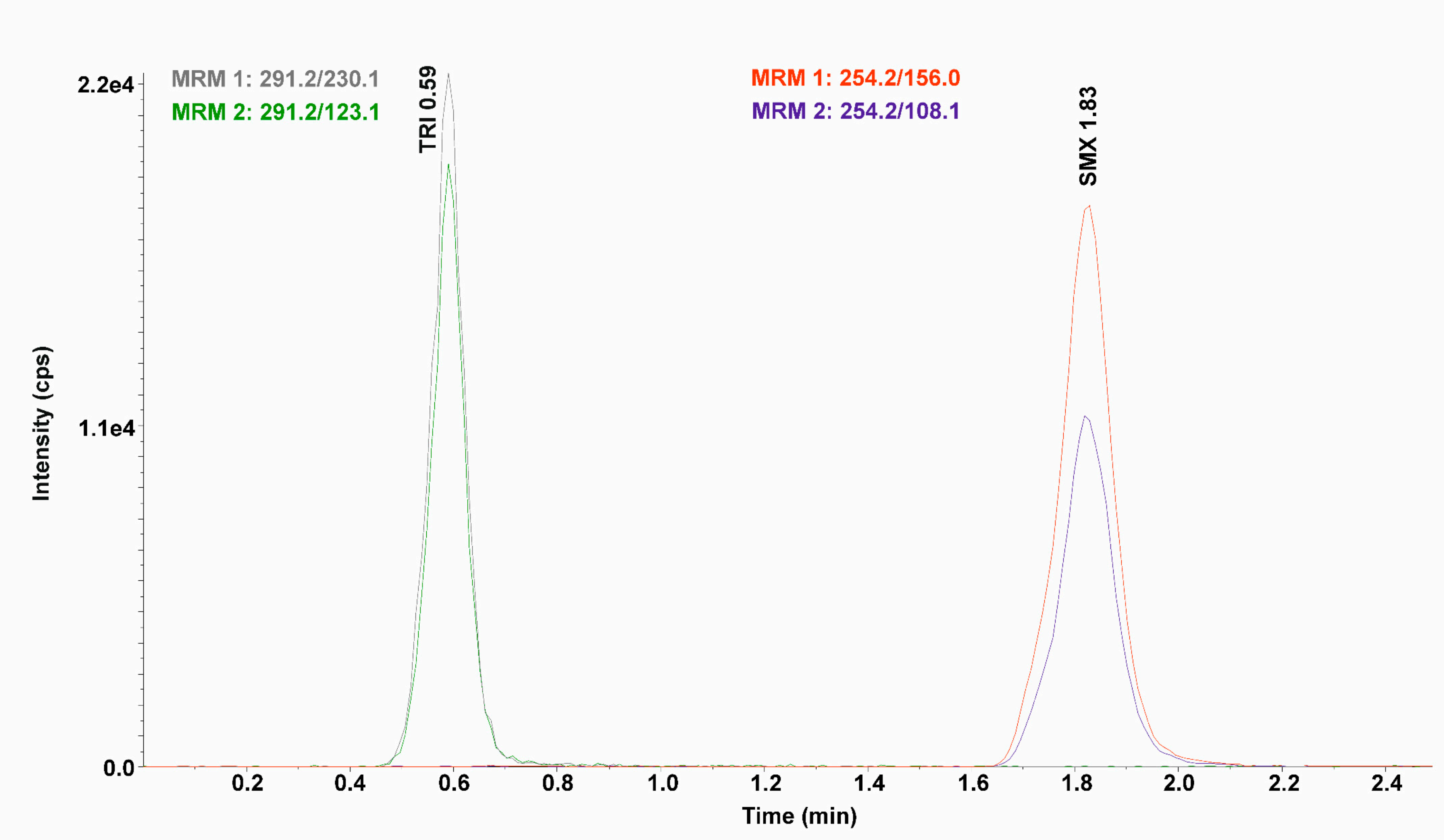

Figure S1. *Representative LC-MS/MS chromatograms of the [M+H]$^+$ions: TRI (m/z 291.2), (2) SMX (m/z 254.2)*

References

1. Anquandah, G.A.K.; Sharma, V.K.; Knight, D.A.; Batchu, S.R.; Gardinali, P.R. Oxidation of Trimethoprim by Ferrate(VI): Kinetics, Products, and Antibacterial Activity. *Environ Sci Technol* **2011**, *45*, 10575–10581, doi:10.1021/es202237g.

2. Lecours, M.-A.; Eysseric, E.; Yargeau, V.; Lessard, J.; Brisard, G.; Segura, P. Electrochemistry-High Resolution Mass Spectrometry to Study Oxidation Products of Trimethoprim. *Environments* **2018**, *5*, 18, doi:10.3390/environments5010018.

3. Eichhorn, P.; Ferguson, P.L.; Pérez, S.; Aga, D.S. Application of Ion Trap-MS with H/D Exchange and QqTOF-MS in the Identification of Microbial Degradates of Trimethoprim in Nitrifying Activated Sludge. *Anal Chem* **2005**, *77*, 4176–4184, doi:10.1021/ac050141p.

4. Ji, Y.; Xie, W.; Fan, Y.; Shi, Y.; Kong, D.; Lu, J. Degradation of Trimethoprim by Thermo-Activated Persulfate Oxidation: Reaction Kinetics and Transformation Mechanisms. *Chemical Engineering Journal* **2016**, *286*, 16–24, doi:10.1016/j.cej.2015.10.050.

5. Alharbi, S.K.; Kang, J.; Nghiem, L.D.; van de Merwe, J.P.; Leusch, F.D.L.; Price, W.E. Photolysis and UV/H2O2 of Diclofenac, Sulfamethoxazole, Carbamazepine, and Trimethoprim: Identification of Their Major Degradation Products by ESI–LC–MS and Assessment of the Toxicity of Reaction Mixtures. *Process Safety and Environmental Protection* **2017**, *112*, 222–234, doi:10.1016/j.psep.2017.07.015.

6. Jia, Y.; Khanal, S.K.; Zhang, H.; Chen, G.H.; Lu, H. Sulfamethoxazole Degradation in Anaerobic Sulfate-Reducing Bacteria Sludge System. *Water Res* **2017**, *119*, 12–20, doi:10.1016/j.watres.2017.04.040.

7. Zheng, L.; Jin, H.; Yu, M.; Zhongwei, Q.; Zhang, L.; Shikun, C.; Li, Z. Degradation of Sulfamethoxazole by Electrochemically Activated Persulfate Using Iron Anode. *International Journal of Chemical Reactor Engineering* **2019**, *17*, 1–14, doi:10.1515/ijcre-2018-0160.

8. Gao, S.; Zhao, Z.; Xu, Y.; Tian, J.; Qi, H.; Lin, W.; Cui, F. Oxidation of Sulfamethoxazole (SMX) by Chlorine, Ozone and Permanganate-A Comparative Study. *J Hazard Mater* **2014**, *274*, 258–269, doi:10.1016/j.jhazmat.2014.04.024.

9. Kim, H.Y.; Kim, T.H.; Cha, S.M.; Yu, S. Degradation of Sulfamethoxazole by Ionizing Radiation: Identification and Characterization of Radiolytic Products. *Chemical Engineering Journal* **2017**, *313*, 556–566, doi:10.1016/j.cej.2016.12.080.

10. Gómez-Ramos, M. del M.; Mezcua, M.; Agüera, A.; Fernández-Alba, A.R.; Gonzalo, S.; Rodríguez, A.; Rosal, R. Chemical and Toxicological Evolution of the Antibiotic Sulfamethoxazole under Ozone Treatment in Water Solution. *J Hazard Mater* **2011**, *192*, 18–25, doi:10.1016/j.jhazmat.2011.04.072.
